# Thymic-Shared Antigen-1 (TSA-1) A Lymphostromal Cell Membrane Ly-6 Superfamily Molecule with a Putative Role in Cellular Adhesion

**DOI:** 10.1155/1998/53157

**Published:** 1998

**Authors:** Brendan J. Classon, Richard L. Boyd

**Affiliations:** 1 Walter & Eliza Hall Institute of Medical Research PO RMH Victoria 3050 Australia; 2 Department of Pathology and Immunology Monash University Medical School Commercial Road Prahran Victoria 3181 Australia

**Keywords:** Membrane protein, Ly-6 superfamily, cell adhesion, thymocyte

## Abstract

The seeding and colonization of the thymus by bone marrow stem cells and the maturation of these cells into mature T lymphocytes are dependent on cell-surface recognition events between different cell lineages within the thymic microenvironment. Positive and negative selection processes within the thymus produce a peripheral T-cell repertoire capable of recognizing peptides derived from foreign antigen bound to self MHC molecules. In addition to the TCR/ MHC-peptide interaction, many other cell-surface molecules act in concert to regulate the kinetics of cellular interactions and intracellular signaling events during thymopoiesis. We have investigated the complexity of the thymic stroma by using monoclonal antibodies to clone cellmembrane molecules of thymic stromal cells. Thymic-shared antigen-1 (TSA-1) is a molecule of interest because it is expressed by both immature thymocytes and stromal cells. We report herein the structural and evolutionary relationships between TSA-1 and molecules of the Ly-6 superfamily (Ly-6SF), and present evidence that TSA-1 functions as a cell-surface receptor by binding a cognate cell target molecule on the surface of a subset of thymocytes.


*Developmental Immunology*, 1998, Vol. 6, pp. 149–156
Reprints available directly from the publisher
Photocopying permitted by license only

© 1998 OPA (Overseas Publishers Association)
N.V. Published by license under the
Harwood Academic Publishers imprint,
part of the Gordon and Breach Publishing Group
Printed in Malaysia

### Thymic-Shared Antigen-1 (TSA-1)

### A Lymphostromal Cell Membrane Ly-6 Superfamily Molecule with a Putative Role in Cellular Adhesion

BRENDAN J. CLASSON[a,b*] and RICHARD L. BOYD[b]

[a]*Walter & Eliza Hall Institute of Medical Research, PO RMH, Victoria, 3050, Australia*; [b]*Department of Pathology and Immunology, Monash University Medical School, Commercial Road, Prahran, Victoria, 3181, Australia*

(*Received 16 June 1997; In final form 19 June 1997*)

The seeding and colonization of the thymus by bone marrow stem cells and the maturation of these cells into mature T lymphocytes are dependent on cell-surface recognition events between different cell lineages within the thymic microenvironment. Positive and negative selection processes within the thymus produce a peripheral T-cell repertoire capable of recognizing peptides derived from foreign antigen bound to self MHC molecules. In addition to the TCR/MHC-peptide interaction, many other cell-surface molecules act in concert to regulate the kinetics of cellular interactions and intracellular signaling events during thymopoiesis. We have investigated the complexity of the thymic stroma by using monoclonal antibodies to clone cell-membrane molecules of thymic stromal cells. Thymic-shared antigen-1 (TSA-1) is a molecule of interest because it is expressed by both immature thymocytes and stromal cells. We report herein the structural and evolutionary relationships between TSA-1 and molecules of the Ly-6 superfamily (Ly-6SF), and present evidence that TSA-1 functions as a cell-surface receptor by binding a cognate cell target molecule on the surface of a subset of thymocytes.

*Keywords:* Membrane protein, Ly-6 superfamily, cell adhesion, thymocyte

### INTRODUCTION

Cell-adhesion mechanisms are responsible for determining the overall tissue architecture within a particular organ. Plasma membrane molecules are major regulators of adhesion events in cellular interactions and they are also responsible for transduction of external stimuli into the cytoplasm. Cell-adhesion receptors are usually members of the immunoglobulin, integrin, and selectin superfamilies, which can interact with other adhesion receptors on adjacent cells or with components of the extracellular matrix (Gumbiner, 1996). Within the thymus, normal cellular differentiation requires interactions between developing thymocytes and stromal cells, a complex network of cell types, including epithelial cells, macrophages,

*Corresponding author. Current address: Dept Pathology and Immunology Monash University Medical School, Commercial Road, Prahran, Victoria, 3181, Australia and dendritic cells (Boyd et al., 1993). The interaction between thymic stromal cells and thymocytes is bidirectional, and the development and function of the thymic stroma and thymocytes themselves are interdependent (Ritter and Boyd, 1993). Thymic-shared antigen-1 (TSA-1) was initially defined as a cell-surface molecule expressed on both immature thymocytes and thymic stromal cells (Godfrey et al., 1992). TSA-1 is also expressed at most stages of B lymphopoiesis, thymic dendritic cells, and activated T cells (Godfrey et al., 1992; Vremec et al., 1992; Kosugi et al., 1994). Monoclonal antibodies (mAbs) to TSA-1 have been shown to block an *in vitro* model of thymopoiesis, fetal thymus organ culture, or FTOC (MacNeil et al., 1993; Randle et al., 1993), TcR-mediated thymocyte apoptosis (Noda et al., 1996), and mature T-cell responses to antigen (Kosugi et al., 1994; Saitoh et al., 1995). This suggests a role for TSA-1 as a cell-surface adhesion molecule in T cells, with an associated signal-transduction role, possibly in conjunction with the TcR complex. We have undertaken cDNA and genomic sequence analysis to reveal evolutionary relationships between TSA-1 and other proteins of the Ly-6SF. We have also investigated whether TSA-1 may function as a receptor by expressing a soluble form of the molecule as a fusion protein, and using this reagent to identify putative cell-surface ligands.

### RESULTS

### Sequence Homology and Genomic Structure

TSA-1 is a cell-surface protein comprising a single domain of 81 amino acids, which is membrane-anchored by a C-terminal GPI moiety (MacNeil et al., 1993; Classon and Coverdale, 1994). TSA-1 is a member of the Ly-6SF, a family of cell-surface and soluble proteins defined on the basis of sequence homology (Gumley et al., 1995a; Palfree et al., 1996). TSA-1 and several representative Ly-6SF sequences are shown aligned in Figure 1A. The data highlight the structural similarities among TSA-1, Ly-6C, and Ly-6A/E, in particular the conservation of 10 Cys residues in the single extracellular domain. Also notable is the Cys-Asn dipetide at or near the C terminus for all molecules. Also shown in Figure 1A are the postsynaptic $\alpha$-cobratoxin and CD59, which share a similar two- and three-stranded $\beta$-sheet structure, and are themselves members of the Ly-6SF (Kieffer et al., 1994). The cobratoxin sequence is different in that only 8/10 Cys residues are conserved,

| | Exon 1 | Intron 1 | Exon 2 | Intron 2 | Exon 3 | Intron 3 | Exon 4 |
|---|---|---|---|---|---|---|---|
| TSA-1 | 68 | 2059 | 101 | 391 | 120 | 104 | 839 |
| ThB | ND | ND | 66 | 672 | 99 | 162 | 533 |
| Ly-6A/E | 53 | 215 | 98 | 961 | 117 | 999 | 601 |
| Ly-6C | 53 | 202 | 99 | 828 | 117 | 1874 | 613 |
| Ly-6F | 60 | 218 | 98 | ≈1300 | 117 | ≈1750 | >490 |
| Ly-6G | 60 | 218 | 98 | 1019 | 117 | ≈1700 | 603 |
| CD59 | 45 | ≈14000 | 85 | ≈5000 | 102 | ≈7000 | >1650 |

FIGURE 1 (A) Sequence homology between TSA-1 and the Ly-6SF. The sequence of TSA-1 is shown aligned with four other Ly-6SF molecules: Ly-6A (LeClair et al., 1986), Ly-6C (Palfree et al., 1988), CD59, and $\alpha$-cobratoxin. (B) Gene structure of TSA-1. The intron/exon structure is drawn to scale for TSA-1 (Classon and Coverdale, 1996). The intron and exon sizes (in nucleotides) for single-domain Ly-6SF molecules whose gene sequences are known are indicated. The gene sequences were obtained from Ly-6A/E (Palfree et al., 1988); Ly-6C (Bothwell et al., 1988); ThB (Gumley et al., 1995b); Ly-6F and Ly-6G (Fleming et al., 1993); and CD59 (Petranka et al., 1992).

but the three-dimensional structure reveals a similar protein folding pattern to CD59. In the mouse, most Ly-6SF genes map to chromosome 15 (LeClair et al. 1987, Kaimura et al., 1992), and in humans, several Ly-6SF genes have been mapped to chromosome 8q24.3-qter (Brakenhoff et al., 1995; Furuhata et al., 1996), which is syntenic with mouse chromosome 15. The genomic organization of TSA-1 (Classon and Coverdale, 1996) reveals the characteristic intron/ exon structure as seen for other members of the Ly-6SF (Figure 1B). The exon sizes for the Ly-6SF genes (Figure 1B) are very similar, suggesting that all Ly-6SF genes derived from a common ancestral precursor by gene duplication.

### Production of a Soluble TSA-1 Fusion Protein

TSA-1 has a single extracellular protein domain with a C-terminal GPI anchor. The GPI anchor signal sequence is characteristically a sequence of hydrophobic amino acids at the C terminus of the protein-coding region, which directs attachment of a GPI anchor (Ferguson and Williams, 1988). In order to express a soluble form of TSA-1, we generated a chimeric cDNA in which the TSA-1 GPI signal sequence was replaced with a short cDNA segment, encoding 23 amino acids of the extracellular "hinge" region of the rat CD8$\alpha$ chain, followed by a translation stop codon (Figure 2A). This segment of CD8 contains O-linked glycans and facilitates expression of protein domains that are otherwise not expressed (Classon et al., 1992). A mouse anti-rat CD8 mAb (OX8) specific for a sialic-acid-dependent epitope contained within this 23 amino acid segment was used (1) to detect fusion proteins and (2) to create a bivalent complex prior to screening for putative binding proteins (see what follows). The chimeric cDNA was ligated into the expression plasmid pEE6 and transfected into CHO-K1 cells. Soluble sTSA-1/CD8 fusion protein was detected by ELISA (Figure 2B) and was found to react with two different anti-TSA-1 mAbs, suggesting the material was expressed in native form. Fusion protein was purified from CHO-cell supernatant by immunoaffinity chromatography using an MTS-35 mAb column. The immunopurified material eluted from the affinity column was judged to be a homogeneous monomer by SDS-PAGE (Figure 2C). Immunopurified sTSA-1 eluted from the MTS-35 column retained antigenicity, as determined by ELISA, and migrated as a monomer by gel filtration (data not shown). Expression levels were estimated by UV spectroscopy (assuming $\epsilon_{1\%/1cm}$ = 10) and silver staining by SDS-PAGE to be approximately 0.2 mg/l.

### Identification of a Putative TSA-1 Cell-Surface Ligand

The presence of TSA-1 on the cell surface of immature thymocytes and thymic stromal elements suggested that either a cell surface or a soluble ligand exists for TSA-1. To investigate the former possibility, sTSA-1/CD8 fusion protein was used as a labeling reagent to identify putative cell-surface ligand(s). The fusion protein was crosslinked with biotinylated OX8 mAb (see Materials and Methods) prior to incubating the complex with target cells. After washing, the bound fusion protein/mAb complex was detected with a streptavidin-phycoerythrin conjugate. As shown in Figure 3, approximately 10% of thymocytes showed weak, but significant staining over background levels (Figure 3, left panel). The negative control for the labeling experiment was a single T-cell receptor V$\alpha$8 chain/CD8 fusion protein, which showed no significant binding. The binding of sTSA-1 to the cell surface was lower than the levels seen for a biotinylated anti-CD8$\beta$ mAb, 53.5 (Figure 3, right panel), and the most likely explanation for this is that sTSA-1 is binding to its target structure with relatively low affinity. Similar experiments with lymph-node cells failed to show significant binding of the sTSA-1 fusion protein. Further experiments to identify lymphoid and stromal-cell lines capable of binding the TSA-1/CD8 fusion protein are in progress.

### DISCUSSION

Molecules of the Ly-6SF are differentially expressed throughout haematopoiesis, suggesting an important role for these molecules in the development and function of the immune system. Within the thymus, TSA-1 is expressed throughout T-cell development until the mature, single-positive cell stage, at which point expression is downregulated (Godfrey et al., 1992). TSA-1 is also expressed on thymic stromal elements, and it is possible that TSA-1 is important in the organization of epithelial-cell networks and interactions between lymphocytes and stromal cells. Blockade of FTOC with a TSA-1 mAb arrests T-cell development just prior to the CD4$^+$CD8$^+$ stage, suggesting an important function for TSA-1 in thymopoiesis (MacNeil et al., 1993; Randle et al., 1993). TSA-1 may be involved in signal transduction because TSA-1 mAbs block TcR-mediated apoptosis in thymocytes (Noda et al., 1996) and inhibit IL-2 release by activated T cells (Saitoh et al., 1995). The results presented herein show that sTSA-1 fusion protein binds to a subset of thymocytes, consistent with the presence of a TSA-1 ligand on these cells. It is possible that the interaction between TSA-1 and its ligand is disrupted when TSA-1 mAbs are added to

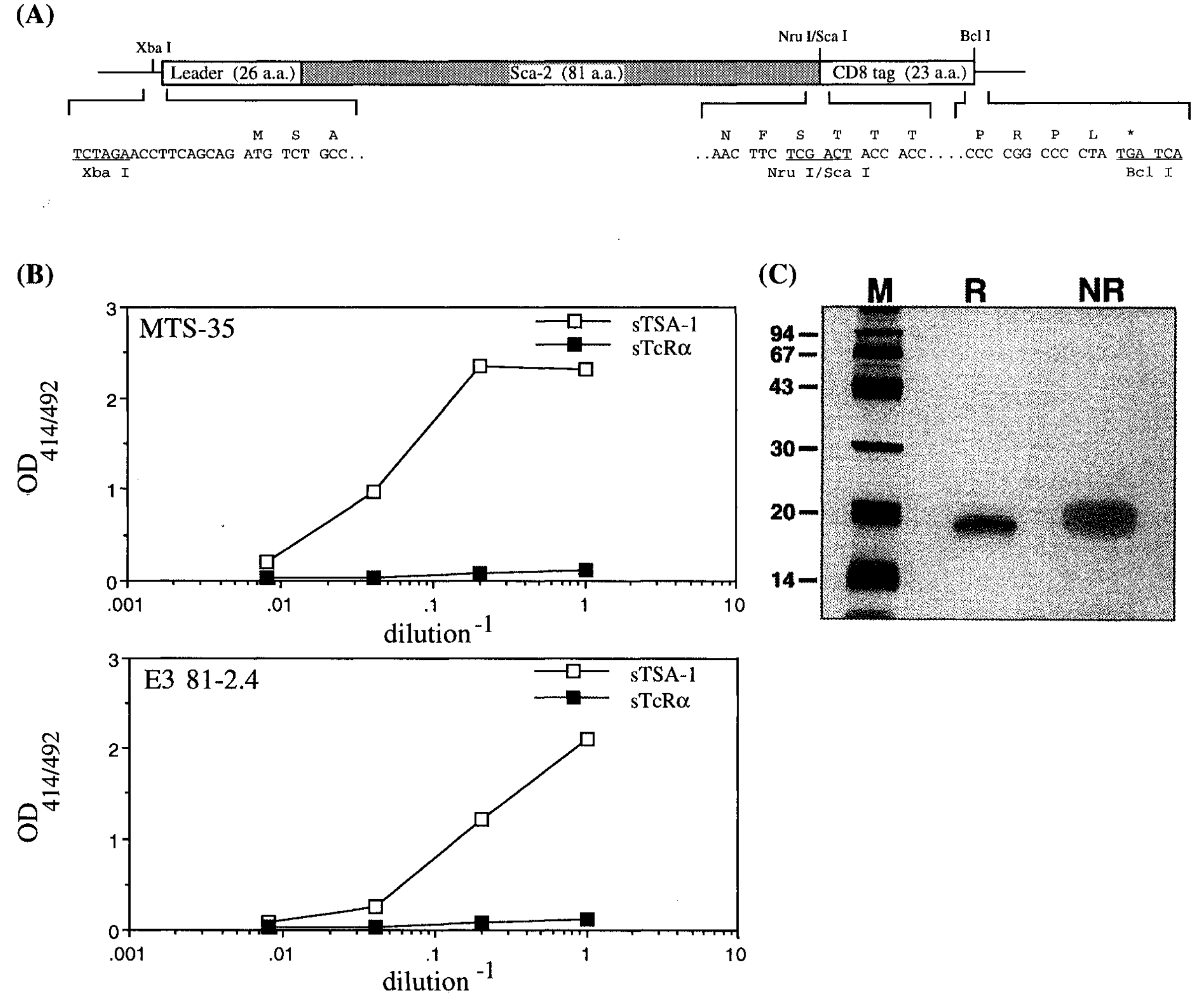

FIGURE 2 Expression of TSA-1/CD8 fusion protein. (A) The TSA-1 cDNA corresponding to the extracellular domain (Classon and Coverdale, 1994) was assembled and expressed in CHO cells as a CD8 fusion protein. (B) ELISA of sTSA-1/CD8 fusion protein with two different TSA-1 mAbs, MTS-35 and E3 81-2.4, as solid-phase capture reagents. (C) SDS-PAGE of immunopurified sTSA-1/CD8 fusion protein. Molecular weight markers (in kilodaltons) are indicated.

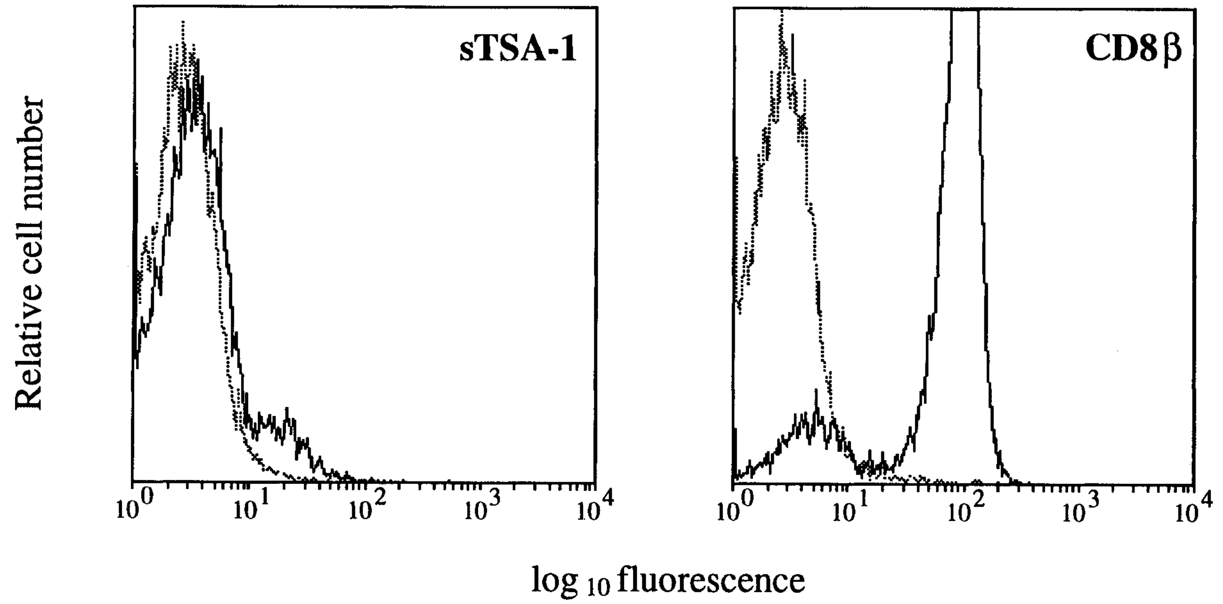

FIGURE 3 A putative cell-surface ligand for TSA-1 expressed on a subpopulation of thymocytes. Thymocytes were labeled with a preformed complex of sTSA-1 and OX8-biotin (see Materials and Methods), and the bound complex was detected with phycoerythrin-conjugated streptavidin.

FTOC, giving rise to blockade of development prior to the CD4+CD8+ stage. An alternative explanation is that the TSA-1 mAb cross-links TSA-1 at the cell surface and delivers a negative signal to the developing T cell. This negative signal may reflect normal TSA-1 function, or it may be an artifactual signal due to receptor cross-linking with a multivalent, high-affinity mAb ligand. Attempts to inhibit FTOC with anti-TSA-1 Fab fragments and soluble TSA-1 fusion protein are currently underway. These reagents are monovalent and would not be expected to trigger an aberrant response by cross-linking surface receptors nor induce antigen loss by capping.

Another Ly-6SF molecule, Ly-6A/E, is also expressed on early thymocytes and thymic stromal cells, but unlike TSA-1, it is also expressed on multipotential bone marrow stem cells (hence its alternative name, stem-cell antigen-1, Sca-1). Ly-6A/E is expressed on early thymocyte precursors, downregulated as thymocytes develop into the CD4+CD8+ stage, and then reexpressed on mature thymocytes (Yeh et al., 1986; Spangrude et al., 1988). Pan-thymic overexpression of Ly-6A/E as a transgene with a CD2 promoter results in a block of T-cell differentiation at the CD4+CD8+ stage, where Ly-6A/E expression is normally downregulated (Bamezai et al., 1995). This result shows the importance of precise regulation of Ly-6A/E expression during thymopoiesis, and overexpressing Ly-6A/E may deliver a dominant negative signal to the thymocyte, possibly related to the mAb induced *in vitro* blockade of FTOC with TSA-1 mAbs (Randle et al., 1993). Ly-6A/E mAbs have been shown to block IL-2 release by activated T cells, in similar fashion to TSA-1 mAbs (Codias et al., 1990; Izon et al., 1996). Given their sequence homology, it is possible that Ly-6A/E has a similar function to TSA-1, despite their different patterns of tissue expression.

Ly-6SF molecules were initially defined as differentiation antigens of lymphocytes. It is now clear that Ly-6SF molecules are not restricted to lymphoid tissues. Ly-6A/E, for example, is expressed at high levels on tubular epithelium and vascular endothelium in the kidney (Blake et al., 1993), and in vasculature of the heart, brain, and liver (van de Rijn et al., 1989). TSA-1 is also expressed in kidney and liver (Classon and Coverdale, 1996). In mice, ThB is expressed in thymocytes and B cells (Gumley et al.,

1995b), whereas in humans, ThB is apparently not expressed in lymphocytes, but is found as a component of desmosomes, where it is known as desmoglein III/dg 4 (Quak et al., 1990; Schrijvers et al., 1991; Brakenhoff et al., 1995). Desmosomes are intercellular organelles found at cell junctions and that mediate adhesion between epithelial cells in many tissues. ThB is localized to the midline of desmosomes, suggesting a structural role in desmosome formation and intercellular adhesion (Quak et al., 1990). Given the structural similarities and similar tissue distribution of ThB and TSA-1, it will be of interest to investigate whether TSA-1 or any other Ly-6SF molecules are also expressed in desmosomes.

Structural similarities suggested by sequence homology points to related functions for Ly-6SF molecules. Four members of the family have well-defined function, namely, the postsynaptic neurotoxins, the urokinase plasminogen activator (uPA) receptor, the phospholipase A2 inhibitor, and CD59. Each of these proteins has evolved to bind target structures, although the nature of the target molecules vary significantly. The neurotoxins of snake venoms are well-characterized inhibitors of neuromuscular transmission that act by binding nicotinic acetylcholine receptors (Endo and Tamiya, 1987). uPAR provides the cell with proteolytic potential for degradation of the extracellular matrix important for cell migration, and is also capable of interacting with and modifying the function of activated integrin molecules (Grondahl-Hansen et al., 1988; Stefansson and Lawrence, 1996; Wei et al., 1996). A phospholipase A2 inhibitor found in snake plasma affords host protection from phospholipase A2, a major degradative enzyme component of many venoms (Fortes-Dias et al., 1994). CD59 protects host cells from autologous lysis by inhibiting formation of the terminal membrane attack complex of complement (Davies and Lachmann, 1993).

Several studies have shown a possible role for Ly-6SF molecules in lymphocyte-cell adhesion. Evidence reported herein suggests that TSA-1 is a cell-surface receptor capable of interacting with a target ligand on the surface of thymocytes. Cell-aggregation assays have shown that Ly-6A/E also binds a heterologous ligand on the surface of lymphoid cells (Bamezai and Rock, 1995), Ly-6C may also function as an adhesion molecule in CTL-target cell interactions (Johnson et al., 1993), and ThB has been shown to be involved in cell-adhesion phenomena (Schrijvers et al., 1991). At present, the molecular nature of these ligands is unclear, and it is conceivable that several molecules share the same ligand. Further experiments involving gene-deficient mice and soluble fusion proteins will clarify the role of the Ly-6SF molecules, and their putative ligands, in tissue organization and adhesion events governing signal transduction and cellular differentiation.

### MATERIAL AND METHODS

### Sequence Alignments

The sequences shown in Figure 1 were aligned using the Multiple Sequence Alignment program at Washington University (http://www.ibc.wustl.edu/msa.html).

### Soluble Fusion Protein and ELISA

A chimeric cDNA comprising the extracellular domain of TSA-1 and the N-terminal leader sequence (codons −26 to +81; Classon and Coverdale, 1994) joined to the rat CD8 hinge sequence was made according to previously published methods (Classon et al., 1992), except that only the first 23 amino acids of the 46 amino acid rat CD8 hinge sequence was used. Fusion protein expressed in CHO-cell supernatant was quantitated by ELISA. Briefly, PVC plates were coated with 25 $\mu$g/ml of either TSA-1-specific mAb (MTS-35 or E3 81-2.4). After blocking with BSA, plates were incubated with titrated amounts of tissue-culture supernatant, washed, and then reacted with biotinylated OX8 mAb, which recognizes the C terminus of the TSA-1 fusion protein. After washing, the wells were then incubated with an appropriate dilution of streptavidin-HRPO conjugate (Dako) and then developed with 2,2′-azino-bis(3-ethylbenzthiazoline-6-sulphonic acid, diammonium salt (ABTS; Sigma) and read in an ELISA plate reader (Titretek).

### Cell-Surface Labeling and FACS Analysis

Single-cell suspensions of thymocytes and lymph-node cells were prepared and labeled with a pre-formed complex of sTSA-1/CD8 fusion protein and biotinylated OX8 mAb. The fusion protein:mAb ratio was determined by inhibition ELISA. Briefly, titrated amounts of sTSA-1/CD8 fusion protein were mixed with a fixed amount of OX8-biotin, such that a 50% inhibition of OX8-biotin binding to immobilized sTSA-1/CD8 was achieved. A second fusion protein, containing a single TcR V$\alpha$8 domain fused to the CD8 hinge sequence, was used as a negative staining control at the same concentration as the sTSA-1 fusion protein. In each case, bound fusion protein was detected using phycoerythrin-conjugated streptavidin (Caltag) and cells were analyzed using a FACScan (Becton Dickinson).

### *Acknowledgments*

This work was supported by the National Health and Medical Research Council, Australia.

### *References*

Bamezai A., Palliser D., Berezovskaya A., McGrew J., Higgins K., Lacy E. and Rock K.L. (1995) Regulated expression of Ly-6A.2 is important for T cell development. *J. Immunol.*, **154**: 4233-4239.

Bamezai A. and Rock K.L. (1995) Overexpressed Ly-6A.2 mediates cell-cell adhesion by binding a ligand expressed on lymphoid cells. *Proc. Natl. Acad. Sci. USA*, **92**: 4294-4298.

Blake P.G., Madrenas J. and Halloran P.F. (1993) Ly-6 in kidney is widely expressed on tubular epithelium and vascular endothelium and is up-regulated by interferon gamma. *J. Amer. Soc. Nephrol.*, **4**: 1140-1150.

Bothwell A., Pace P.E. and LeClair K.P. (1988) Isolation and expression of an IFN-responsive *Ly-6C* chromosomal gene. *J. Immunol.*, **140**: 2815-2820.

Boyd R.L., Tucek C.L., Godfrey D.I., Izon D.J., Wilson T.J., Davidson N.J., Bean A.G.D., Ritter M. and Hugo P. (1993) The thymic microenvironment. *Immunol. Today*, **14**: 445-459.

Brakenhoff R.H., Gerretsen M., Knippels E.M.C., van Dijk M., van Essen H., Weghuis D.O., Sinke R.J., Snow G.B. and van Dongen G.A.M.S. (1995) The human E48 antigen, highly homologous to the murine Ly-6 antigen ThB, is a GPI-anchored molecule apparently involved in keratinocyte cell-cell adhesion. *J. Cell Biol.*, **129**: 1677-1689.

Classon B.J., Brown M.H., Garnett D., Somoza C., Barclay A.N., Willis A.C. and Williams A.F. (1992) The hinge region of the CD8 alpha chain: Structure, antigenicity, and utility in expression of immunoglobulin superfamily domains. *Int. Immunol.*, **4**: 215-225.

Classon B.J. and Coverdale L. (1994) Mouse stem cell antigen-2 (Sca-2) is a member of the Ly-6 family of cell surface proteins. *Proc. Natl. Acad. Sci. USA*, **91**: 5296-5300.

Classon B.J. and Coverdale L. (1996) Genomic organization and expression of mouse thymic shared antigen-1 (TSA-1): Evidence for a processed pseudogene. *Immunogenetics*, **44**: 222-226.

Codias E.K., Rutter J.E., Fleming T.J. and Malek T.R. (1990) Down regulation of IL-2 production by activation of T cells through Ly-6A/E. *J. Immunol.*, **145**: 1407-1414.

Davies A. and Lachmann P.J. (1993) Membrane defence against complement lysis: The structure and biological properties of CD59. *Immunol. Res.*, **12**: 258-275.

Endo T. and Tamiya N. (1987) Current view on the structure-function relationship of postsynaptic neurotoxins from snake venoms. *Pharmacol. Ther.*, **34**: 403-451.

Ferguson M.A. and Williams A.F. (1988) Cell-surface anchoring of proteins via glycosyl-phosphatidylinositol structures. *Ann. Rev. Biochem.*, **57**: 285-320.

Fleming T.J., O'hUigin C. and Malek T.R. (1993) Characterization of two novel Ly-6 genes. Protein sequence and potential structural similarity to alpha-bungarotoxin and other neurotoxins. *J. Immunol.*, **150**: 5379-5390.

Fortes-Dias C.L., Lin Y., Ewell J., Diniz C.R. and Liu T.-Y. (1994) A phospholipase A2 inhibitor from the plasma of the South American rattlesnake (*Crotalus durissus terrificus*): Protein structure, genomic structure, and mechanism of action. *J. Biol. Chem.*, **269**: 15646-15651.

Furuhata T., Tokino T., Urano T. and Nakamura Y. (1996) Isolation of a novel GPI-anchored gene specifically regulated by p53: Correlation between its expression and anti-cancer drug sensitivity. *Oncogene*, **13**: 1965-1970.

Godfrey D. I., Masciantonio M., Tucek C.L., Malin M.A., Boyd R.L. and Hugo P. (1992) Thymic shared antigen-1: A novel thymocyte marker discriminating immature from mature thymocyte subsets. *J. Immunol.*, **148**: 2006-2011.

Grondahl-Hansen J., Lund L.R., Ralfkiaer E., Ottevanger V. and Dano K. (1988) Urokinase- and tissue-type plasminogen activators in keratinocytes during wound reepithelialization in vivo. *J. Invest. Dermatol.*, **90**: 790-795.

Gumbiner B.M. (1996) Cell adhesion: The molecular basis of tissue architecture and morphogenesis. *Cell*, **84**: 345-357.

Gumley T.P., McKenzie I.F.C. and Sandrin M.S. (1995a) Tissue expression, structure and function of the murine Ly-6 family of molecules. *Immunol. Cell Biol.*, **73**: 277-296.

Gumley T.P., McKenzie I.F.C. and Sandrin M.S. (1995b) Sequence and structure of the mouse ThB gene. *Immunogenetics*, **42**: 221-224.

Izon D.J., Oritani K., Hamel M., Calvo C.R., Boyd R.L., Kincade P.W. and Kruisbeek A.M. (1996) Identification and functional analysis of Ly-6A/E as a thymic and bone marrow stromal antigen. *J. Immunol.*, **156**: 2391-2399.

Johnson R., Lancki D.W. and Fitch F.W. (1993) Accessory molecules involved in antigen-mediated cytolysis and lymphokine production by cytotoxic lymphocyte subsets. I. Identification of functions for the T cell surface molecules Ly-6C and Thy-1. *J. Immunol.*, **151**: 2986-2999.

Kamiura S., Nolan C.M. and Meruelo D. (1992) Long range physical map of the Ly-6 complex: Mapping the Ly-6 multigene family by field inversion and two dimensional gel electrophoresis. *Genomics*, **12**: 89-105.

Kieffer B., Driscoll P.C., Campbell I.D., Willis A.C., van der Merwe P.A. and Davis S.J. (1994) Three-dimensional solution structure of the extracellular region of the complement regulatory protein CD59, a new cell surface protein domain related to snake venom neurotoxins. *Biochemistry*, **33**: 4471-4482.

Kosugi A., Saitoh S., Narumiya S., Miyake K. and Hamaoka T. (1994) Activation-induced expression of thymic shared antigen-1 on T lymphocytes and its inhibitory role for TCR-mediated IL-2 production. *Int. Immunol.*, **6**: 1967-1976.

LeClair K.P., Palfree R.G., Flood P.M. and Hammerling U. (1986) Isolation of a murine Ly-6 cDNA reveals a new multi gene family. *EMBO J.*, **5**: 3227-3234.

MacNeil I., Kennedy J., Godfrey D.I., Jenkins N.A., Masciantonio M., Mineo C., Gilbert D.J., Copeland N.G., Boyd R.L. and Zlotnik A. (1993) Isolation of a cDNA encoding thymic shared antigen-1, a new member of the Ly-6 family with a possible role in T cell development. *J. Immunol.*, **151**: 6913-6923.

Noda S., Kosugi A., Saitoh S., Narumiya S. and Hamaoka T. (1996) Protection from anti-TCR/CD3-induced apoptosis in immature thymocytes by a signal through thymic shared antigen-1/stem cell antigen-2. *J. Exp. Med.*, **183**: 2355-2360.

Palfree R.G.E. (1996) Ly-6-domain proteins—New insights and new members: A C-terminal Ly-6 domain in sperm acrosomal protein SP-10. *Tissue Antigens*, **48**: 71-79.

Palfree R.G.E., Sirlin S., Dumont F.J. and Haemmerling U. (1988) N-terminal and cDNA characterization of murine lymphocyte antigen Ly-6C.2. *J. Immunol.*, **140**: 305-310.

Petranka J.G., Fleenor D.E., Sykes K., Kaufman R.E. and Rosse W.F. (1992) Structure of the CD59-encoding gene: Further evidence of a relationship to the murine lymphocyte antigen Ly-6 protein. *Proc. Natl. Acad. Sci. USA*, **89**: 7876-7879.

Quak J.J., van Dongen G.A.M.S., Balm A.J.M., Brakkee J.P.G., Scheper R.L., Snow G.B. and Meijer C.J.L.M. (1990) A 22-kDa surface antigen detected by monoclonal antibody E48 is exclusively expressed in stratified squamous and transitional epithelia. *Amer. J. Pathol.*, **136**: 191-197.

Randle E.S., Waanders G.A., Masciantonio M., Godfrey D.I. and Boyd R.L. (1993) A lymphostromal molecule, thymic shared Ag-1, regulates early thymocyte development in fetal thymus organ culture. *J. Immunol.*, **151**: 6027-6035.

Ritter M. and Boyd R.L. (1993) The symbiotic developmental relationship between thymic lymphocytes and stromal cells. *Immunol. Today*, **14**: 462-469.

Saitoh S., Kosugi A., Noda S., Yamamoto N., Ogata M., Minami Y., Miyake K. and Hamaoka T. (1995) Modulation of TCR-mediated signaling pathway by thymic shared antigen-1 (TSA-1)/stem cell antigen-2 (Sca-2). *J. Immunol.*, **155**: 5574-5581.

Schrijvers A.H.G.J., Gerretsen M., Fritz J., van Walsum M., Quak J.J., Snow G.B. and van Dongen G.A.M.S. (1991) Evidence for a role of the monoclonal antibody E48 defined antigen in cell-cell adhesion in squamous epithelia and head and neck squamous cell carcinomas. *Exp. Cell Res.*, **196**: 264-269.

Spangrude G.J., Aihara Y., Weissman I.L. and Klein J. (1988) The stem cell antigens SCA-1 and SCA-2 subdivide thymic and peripheral T lymphocytes into unique subsets. *J. Immunol.*, **141**: 3697-3707.

Stefansson S. and Lawrence D.A. (1996) The serpin PAI-1 inhibits cell migration by blocking integrin $\alpha_v\beta_3$ binding to vitronectin. *Nature*, **383**: 441-443.

van de Rijn M., Heimfeld S., Spangrude G.J. and Weissman I.L. (1989) Mouse hematopoietic stem cell antigen Sca-1 is a member of the Ly-6 antigen family. *Proc. Natl. Acad. Sci. USA*, **86**: 4634-4638.

Vremec D., Zorbas M., Scollay R., Saunders D.J., Ardavin C.F., Wu L. and Shortman K. (1992) The surface phenotype of dendritic cells purified from mouse thymus and spleen: Investigation of the CD8 expression by a subpopulation of dendritic cells. *J. Exp. Med.*, **176**: 47-58.

Wei Y., Lukashev M., Simon D.I., Bodary S.C., Rosenberg S., Doyle M.V., Chapman H.A. (1996) Regulation of integrin function by the urokinase receptor. *Science*, **273**: 1551-1555.

Yeh E.T., Reiser H., Benacerraf B. and Rock K.L. (1986) The expression, function and ontogeny of a novel T cell-activating protein, TAP, in the thymus. *J. Immunol.*, **137**: 1232-1238.

